# The Effect of Sacubitril/Valsartan Treatment on Cardiac and Renal Functions of a Patient With Cardiorenal Syndrome Type 4 and Stage 5 CKD After More Than Three Years of Follow-Up

**DOI:** 10.3389/fmed.2022.817833

**Published:** 2022-03-11

**Authors:** Shuiqin Cheng, Tingting Zhou, Le Yu, Yunmin Chen, Zhihong Zhang, Jinquan Wang, Yusheng Yu

**Affiliations:** Department of Nephrology, National Clinical Research Center of Kidney Disease, Jinling Hospital, Nanjing University School of Medicine, Nanjing, China

**Keywords:** sacubitril/valsartan, cardiorenal syndrome type 4, stage 5 CKD, cardiac function, renal function, long-term follow-up

## Abstract

It is difficult to treat cardiorenal syndrome (CRS) in clinical practice, which is the common reason for the death of patients. This report aimed to describe the effects of sacubitril/valsartan treatment on cardiac and renal functions of a patient with cardiorenal syndrome type 4 (CRS4) after more than 3 years of follow-up. A 77-year-old Chinese woman was admitted to our hospital because of CRS4 and stage 5 chronic kidney disease (CKD), who had a history of long-term proteinuria and renal failure. The patient's cardiothoracic ratio (CTR) measured by chest X–ray was 0.6. Cardiac ultrasonography showed that the left ventricular ejection fraction (LVEF) was 0.40. The patient had been treated for heart failure (HF) for 5 months, but there was no improvement in clinical manifestations, and the renal function gradually deteriorated. In our hospital, she received sacubitril/valsartan treatment for at least 40 months. The symptoms of HF relieved, and the indices of cardiac function improved. In addition, the patient's renal function was stable. During the treatment, the dosage of sacubitril/valsartan needed to be adjusted to achieve the optimal therapeutic effect. Follow-up results showed that she achieved cardiac function of New York Heart Association (NYHA) class II with an ejection fraction of 0.60 and E/A > 1 indicated by echocardiogram, and did not develop hyperkalemia. In summary, the improvement of cardiac and renal functions of the CRS4 patient was associated with the long-term sacubitril/valsartan treatment.

## Introduction

Cardiorenal syndrome (CRS) is defined as a complex pathophysiological disorder of the heart and the kidney, in which acute or chronic dysfunction of one organ may induce acute or chronic dysfunction of the other. Due to the interaction between the heart and kidneys, cardiac failure and renal failure may simultaneously occur, forming a vicious cycle. Patients with chronic kidney disease (CKD) are at a high risk of heart failure (HF) (1). CKD may lead to cardiovascular events, such as HF, and pathologic cardiac changes are called cardiorenal syndrome type 4 (CRS4) (2). The treatment of CRS4 is the main challenge because several drugs, which are used to control organ failure, may worsen another organ's function (3). Therefore, further comprehensive and systematic treatment for CRS4 is required according to the pathological mechanisms (4).

Atrial cardiomyocytes secrete atrial natriuretic peptides, B-type natriuretic peptides, and C-type natriuretic peptides, which can bind to the corresponding receptors to produce strong natriuretic, diuretic, and vasodilators, and inhibit activation of Renin-angiotensin-aldosterone system (RAAS) and proliferation of vascular smooth muscle or endothelial cells. Neprilysin can hydrolyze natriuretic peptides. Neprilysin inhibitors can inhibit the hydrolysis of natriuretic peptide by neprilysin and increase the level of natriuretic peptide, which are protective for HF (5). Sacubitril/valsartan is an angiotensin receptor-neprilysin inhibitor (ARNI), which can elevate natriuretic peptide and inhibit angiotensin II to protect cardiac function (6). Recent studies have shown that sacubitril/valsartan can significantly improve the symptoms of HF (7) and stabilize renal function (8). Besides, it can improve the quality of life of patients with CRS. We successfully treated a patient of New York Heart Association (NYHA) class IV HF with a reduced ejection fraction (HFrEF) by sacubitril/valsartan. After long-term follow-up, the stability of the patient's cardiac and renal functions was confirmed.

## Case Description

**Figure 1:**
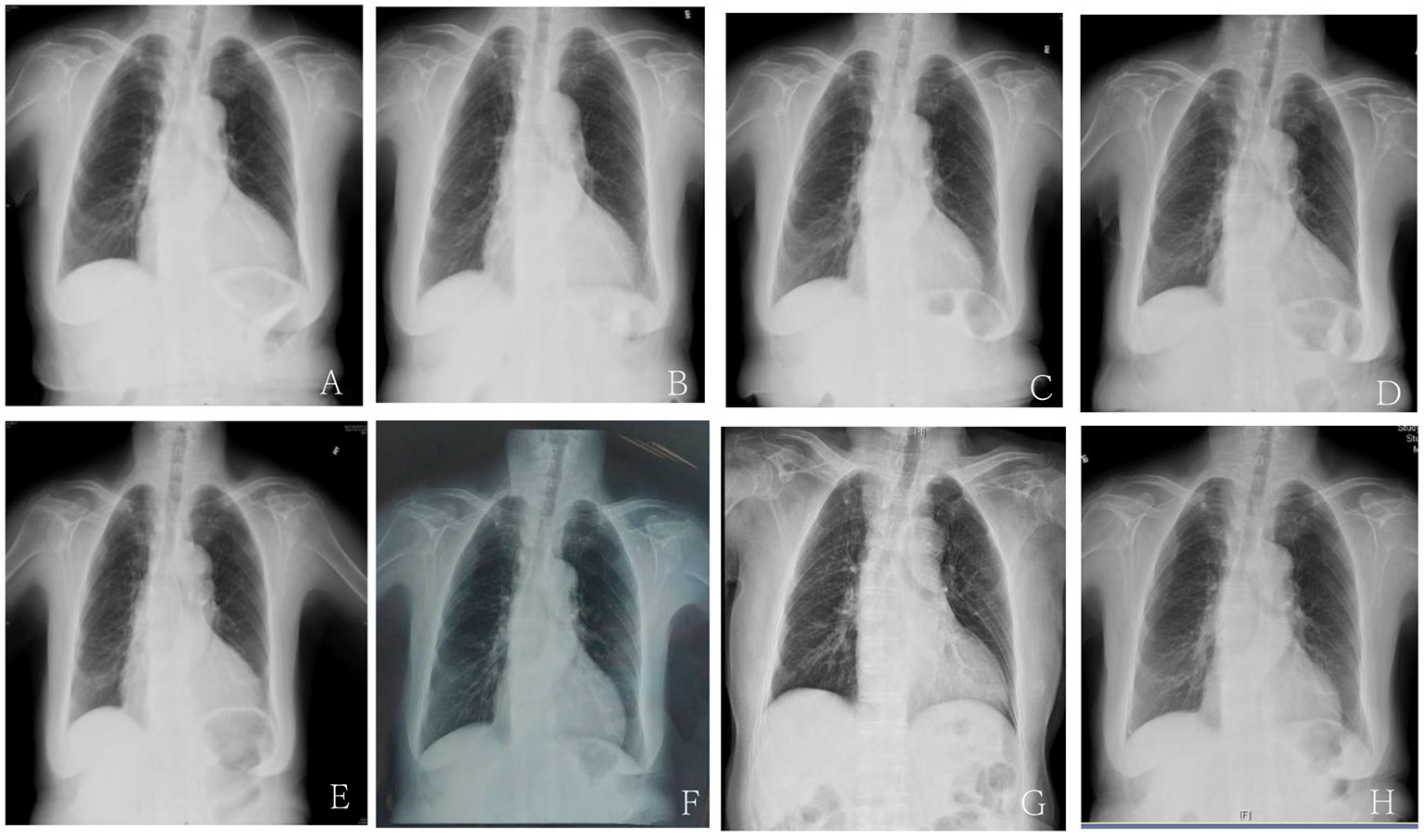
The cardiothoracic ratio (CTR) of chest X-ray before and after treatment with Sacubitril/valsartan. **(A)** The CTR of chest X-ray upon admission due to the first episode of HF before treatment with sacubitril/valsartan was 0.6. **(B)** The CTR of chest X-ray at 2 months after sacubitril/valsartan treatment was 0.57. **(C)** The CTR of chest X-ray at 4 months after sacubitril/valsartan treatment was 0.56. **(D)** The CTR of chest X-ray upon admission due to the second episode of HF 8 months after dosage reduction of sacubitril/valsartan treatment was 0.64. **(E)** The CTR of chest X-ray at 11 months after sacubitril/valsartan treatment was 0.6. **(F)** The CTR of chest X-ray at 17 months after increasing the dosage of sacubitril/valsartan treatment was 0.56. **(G)** The CTR of chest X-ray at 25 months after sacubitril/valsartan treatment was 0.57. **(H)** The CTR of chest X-ray at 40 months after sacubitril/valsartan treatment was 0.55.

**Figure 2:**
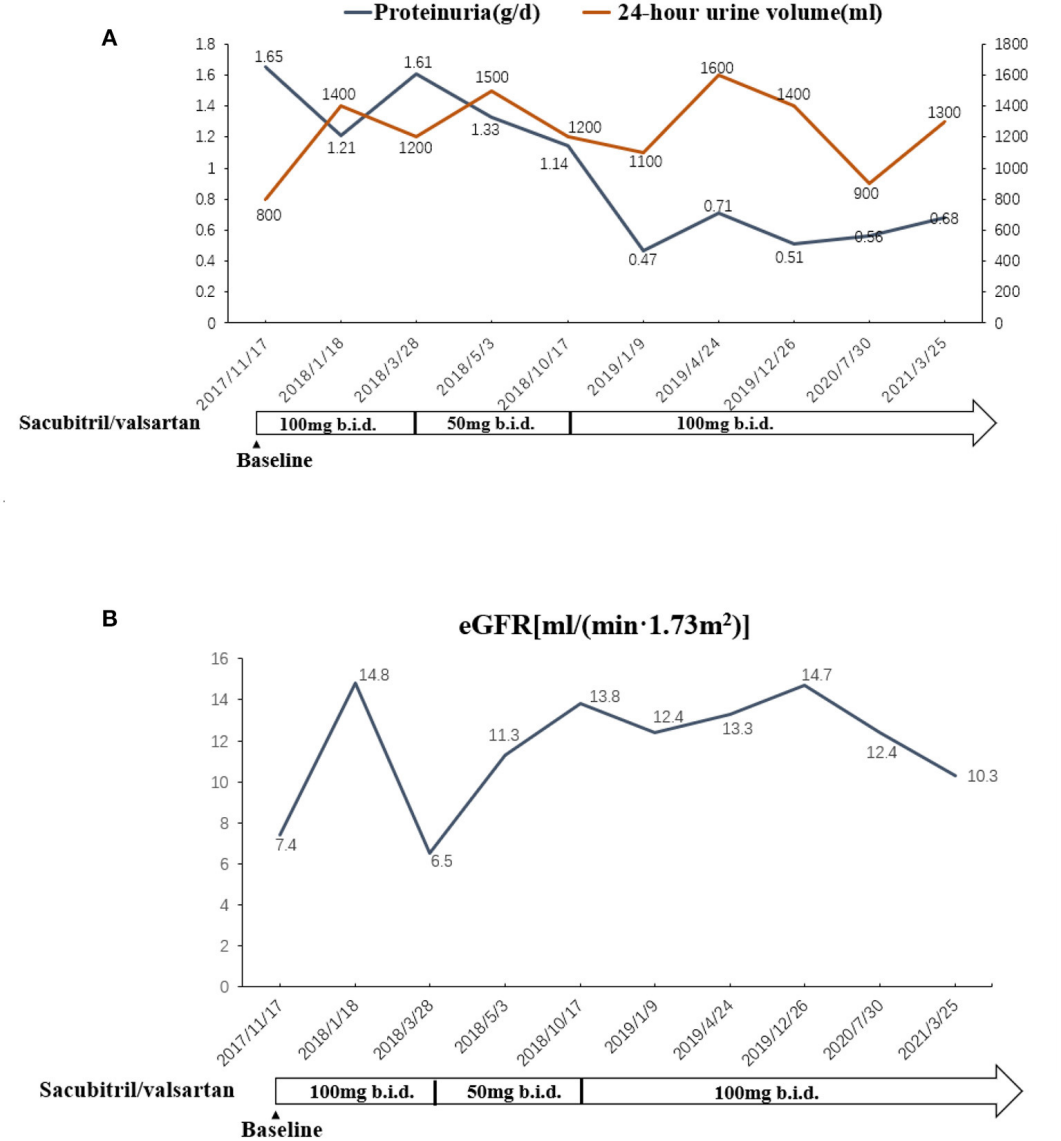
**(A)** The changes of urinary volume and proteinuria in association with the adjustment of sacubitril/valsartan. **(B)** The changes of renal function in association with the adjustment of sacubitril/valsartan.

**Figure 3:**
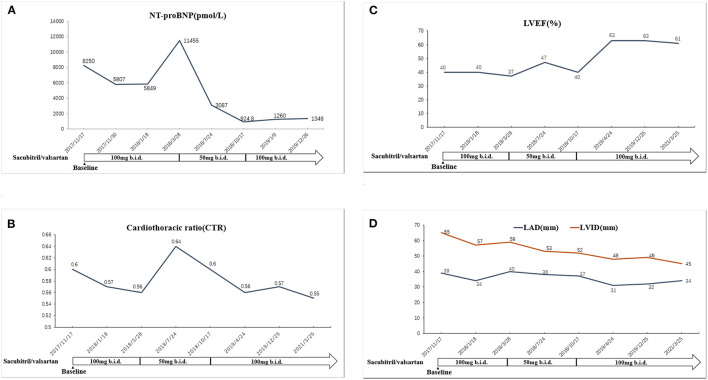
**(A)** The change of NT-proBNP in association with the adjustment of sacubitril/valsartan. **(B)** The change of LVEF in association with the adjustment of sacubitril/valsartan. **(C)** The change of cardiothoracic ratio in association with the adjustment of sacubitril/valsartan. **(D)** The changes of LAD and LVID in association with the adjustment of sacubitril/valsartan. LVEF, left ventricular ejection fraction; LAD, left atrial dimension; LVID, left ventricular internal diameter.

**Figure 4:**
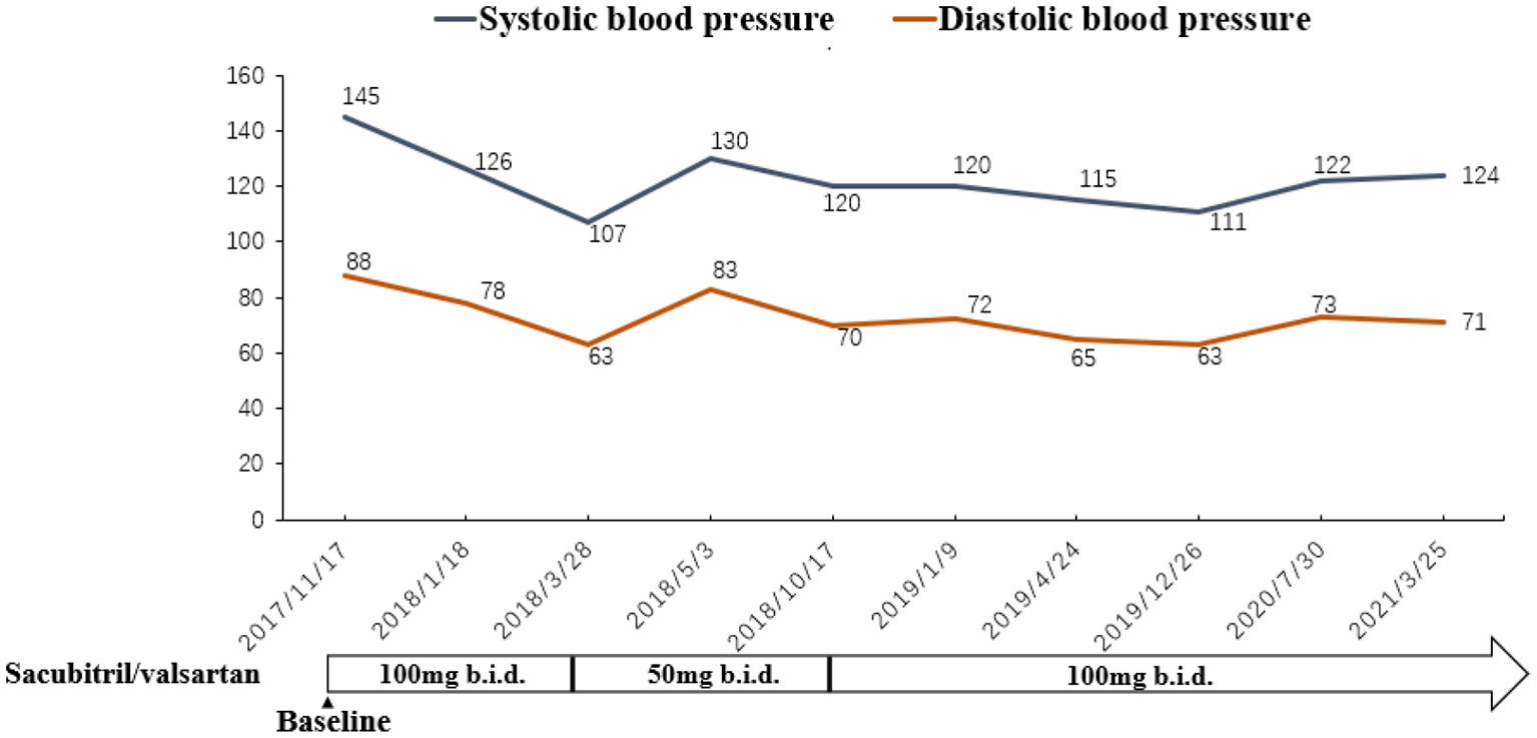
The changes of blood pressure during the treatment of sacubitril/valsartan.

A 77-year-old Chinese woman who had an 11-year history of proteinuria, hypertension, gradually elevated serum creatinine, and renal anemia was investigated. She never underwent renal biopsy, and the possible etiology for the CKD was considered as hypertensive nephropathy or chronic glomerulonephritis. Cardiac function was normal at the beginning of CKD. Long-term antihypertensive therapy was given based on ACEI and correction of renal anemia, and the renal function was stable, and the serum level of creatinine varied from 260 to 300 μmol/L. She was admitted to our hospital in November 2017 with 5 months of HF symptoms, such as chest tightness, orthopnea, wheezing, dyspnea, uncontrollable hypertension, and chest X-ray and cardiac ultrasonography indicated an enlarged heart and HFrEF. The patient's cardiothoracic ratio (CTR) measured by chest X–ray was 0.6. Cardiac ultrasonography showed that her left ventricular ejection fraction (LVEF) was 0.40. The N-terminal pro B-type natriuretic peptide (NT-proBNP) concentration significantly increased (8,250 pmol/L; normal value <125 pmol/L). The cardiac function of this patient was assessed as NYHA class IV. The value of the 6-minute walk test (6MWT) was 185 m. The patient had been treated for HF using such drugs as diuretics, ACEI, β-blockers, aldosterone receptor antagonists, and trimetazidine for 5 months, but there was no improvement in clinical manifestations, and the renal function gradually deteriorated. Upon admission, the serum level of creatinine was 463 μmol/L with an estimated glomerular filtration rate (eGFR) of 7.4 ml/min/1.73 m^2^ at baseline, and the eGFR was evaluated by CKD-EPI equation (9) in this patient. CRS4 was considered based on the patient's medical history and laboratory examinations. There were no other cardiovascular and cerebrovascular complications, such as atrial fibrillation or coronary artery disease. She was prescribed an initial dose (50 mg b.i.d.) of sacubitril/valsartan, rather than ACEI. Two weeks later, it was progressively titrated to the maximum tolerated dose (100 mg b.i.d.). The patient's renal and cardiac functions gradually improved, manifested as decreased serum levels of creatinine, increased urine volume and LVEF. The dose of diuretic was tapered off to 20 mg/d, and it was discontinued because of transient elevation of serum creatinine. The patient's left atrial dimension (LAD), left ventricular internal diameter (LVID), and CTR decreased, and hypertension controlled (i.e., systolic and diastolic blood pressure decreased). Afterward, because of fluctuations in the renal function, the dose of sacubitril/valsartan was decreased to 50 mg b.i.d. However, the patient's indices of cardiac function recovered, and some symptoms of HF appeared. The dose was re-elevated to the maximum (100 mg b.i.d.), and the patient's cardiac function gradually improved again. The renal function was also stabilized. At the time of controlling HF, CTR decreased (Figure 1), residual renal functions such as urinary volume, proteinuria and eGFR were stable (Figure 2), and eGFR was 10.3 ml/min/1.73 m^2^ at the last follow-up. The indices of cardiac function, including NT-proBNP, LVEF, LAD, LVID improved (Figure 3). Systolic and diastolic blood pressure during the treatment of sacubitril/valsartan gradually decreased and stabilized (Figure 4). The dose of sacubitril/valsartan was re-adjusted to the maximum of 100 mg b.i.d., and no harmful side effect was detected in other organs. Follow-up results showed that she achieved cardiac function of NYHA class II with an ejection fraction of 0.60 and E/A > 1 indicated by echocardiogram. The value of 6MWT increased to 410 m. There was no episode of worsening HF, and renal function progressed slowly. With a low potassium diet, this patient did not develop hyperkalemia after treatment with sacubitril/valsartan.

## Discussion

CRS is generally defined as a pathophysiological disorder of the heart and kidney, in which acute or chronic dysfunction of one organ may induce acute or chronic dysfunction of the other. CRS is a common clinical syndrome, and it includes 5 types (type 1, type 2, type 3, type 4, and type 5) (10). Besides, CRS3 (acute cardiac failure caused by acute renal failure) and CRS4 (chronic cardiac failure caused by chronic renal failure) have been frequently observed in patients with kidney disease, and renal failure was a poor prognostic factor of CRS patients (11).

As the first ARNI, sacubitril/valsartan has a unique dual mechanism of action. In addition to inhibiting RAAS, it also specifically inhibits neprilysin, so that the body's natriuretic peptide system can fully promote vasodilation, diuresis, and natriuresis, and it also plays a protective role in coronary artery occlusion, kidney disease, etc. (12). The prognosis of patients with HF can be improved by promising management of neuroendocrine tumors, rather than by traditional treatment (13). In the present case, the dose of sacubitril/valsartan was adjusted to the routine dosage (100 mg b.i.d.) after the initial treatment with a small dose for 2 weeks. Symptoms, such as palpitation and chest tightness significantly controlled, urinary volume increased, and mental and nutritional statuses improved. At the same time, regarding indices of cardiac function, e.g., NT-proBNP, CTR showed a downward trend, and LVEF increased gradually. After 3 months of treatment, the dose of sacubitril/valsartan was reduced to 50 mg b.i.d. due to some factors, including fluctuation of renal function and medication costs. The HF reattack occurred six months later. The dose of sacubitril/valsartan was re-adjusted to 100 mg b.i.d., and the above-mentioned symptoms quickly controlled after 1 week. At the same time, with the control of HF, CTR decreased, residual renal function was stable, and the indices of cardiac function improved. It is proven that inhibition of neprilysin is a new therapeutic target for cardio-renal protection in patients with CKD, which not only improves cardiac function but also significantly restores residual renal function in such patients.

Compared with renin-angiotensin system inhibitors (RASIs), sacubitril/valsartan has been shown to improve cardiac function of patients more effectively and rapidly (14). The PARADIGM-HF study (14) showed that sacubitril/valsartan could reduce the risk of cardiovascular mortality by 20%, and decrease the risk of HF hospitalization by 21%. It could also improve symptoms of HF and patients' quality of life. The incidence of side effects, such as cough, hyperkalemia, and impaired renal function reduced as well. Participants with eGFR ≥30 ml/min/1.73 m^2^ were recruited in the PARADIGM-HF study, and thus the results are also encouraging for those with stage 5 CKD. It is clinically relevant that a patient with stage 5 CKD can respond well to this newer agent, with increased urine output and improvement of both cardiac and renal functions and no concerns about hyperkalemia. Transient elevation of serum creatinine may be associated with a higher dose of sacubitril/valsartan or long-term use of diuretics, and serum creatinine decreased after reduction of sacubitril/valsartan dose and discontinuation of diuretics.

Januzzi et al. (15) demonstrated that sacubitril/valsartan could reduce the patient's serum level of NT-ProBNP and increase LVEF significantly, which is consistent with the results of the present case. In the current case, the patient's severity of CTR decreased gradually after sacubitril/valsartan treatment, indicating that the patient's cardiac tissue structure improved significantly. Previous studies (16) have confirmed that the use of sacubitril/valsartan in patients with LVEF ≤ 40% can remarkably enhance their cardiac function. In recent years, some scholars (17, 18) have shown that the use of sacubitril/valsartan in HF patients with LVEF ≥40% can also markedly improve the NYHA grade. Volpe et al. (19) reported that sacubitril/valsartan could be the first-line therapy for patients with HFrEF or heart failure with mid-range ejection fraction (HFmrEF). In our study, after taking sacubitril/valsartan, LVEF increased gradually. LVEF slowly elevated to 47% at 8 months after taking sacubitril/valsartan, which declined as the dosage was reduced, and gradually rose again as the dosage was increased. The cardiac function improved after sacubitril/valsartan treatment in a dose-dependent manner. At the end of the follow-up, the LVEF was significantly higher than that at the baseline. The value of 6MWT increased, which is in agreement with Beltrán et al.'s findings (20). Additionally, we found that sacubitril/valsartan could reduce the patient's blood pressure. Scholars demonstrated that sacubitril/valsartan could significantly reduce patients' blood pressure, especially systolic blood pressure (21, 22) and night blood pressure (23). Sacubitril/valsartan also decreased diastolic blood pressure (22). It can be used as an option for antihypertensive therapy, especially for patients who suffer from refractory hypertension. It is noteworthy that low-dose sacubitril/valsartan can also significantly improve patients' cardiac function, especially in some elderly, intolerable patients (24).

Abumayyaleh M et al. (25) reported that sacubitril/valsartan could significantly improve the LVEF of patients with HFrEF and decrease the mortality rate as compared with ACEI, and thus the patients should be treated as early as possible to delay the progression of HFrEF. Recent studies showed that sacubitril/valsartan may impact the risk of sudden cardiac death in patients suffering from HFrEF (14, 26–28). Therefore, the effect of sacubitril/valsartan on cardiac outcomes of HF patients is of interest when such data are analyzed.

The excessive production of RAAS may lead to vasoconstriction of arterioles and an increase in blood pressure. The excessive activation of RAAS in the heart and kidney plays an important role in myocardial remodeling, glomerular sclerosis and tubular interstitial fibrosis. Therefore, RASI is a good choice for the treatment of patients with HF and early CKD (29). As cardiac function improved, renal perfusion enhanced, followed by an increased GFR. In addition, compared with traditional RASI, ARNI significantly reduced proteinuria, improved renal ultrastructure, and decreased biomarkers of the renal tubulointerstitial lesion (30, 31), which is independent of blood pressure reduction. The acting mechanisms are considered as inhibition of inflammation and oxidative stress, as well as drug-specific protection of podocyte integrity (30–32).

For some patients with CKD, however, RASI may cause a rapid increase in serum levels of creatinine in a short period due to the reduction of both intraglomerular perfusion pressure and glomerular filtration rate. Although RASI can protect the kidney and heart for a long period, there are still some patients who have to withdraw the therapy because of the rapid increase of creatinine or hyperkalemia after treatment, restricting the clinical application of RASI in CRS (33). Masarone et al. (34) found that the use of sacubitril/valsartan treatment in patients with HFrEF had no significant effect on eGFR after 3 months of therapy compared with the baseline. During the follow-up of 650 ± 80 days, there was no HF-related death, HF-related hospitalization, and need for renal replacement therapy, indicating that sacubitril/valsartan treatment could stabilize renal and cardiac functions. It may be safe and effective to use ARNI therapy even at a very low eGFR for this patient, even at a point when other patients may choose dialysis.

Additionally, the PARADIGM-HF study showed that ARNI could attenuate the risk of hyperkalemia in patients with HF compared with benazepril (15). The patient in the current research did not develop hyperkalemia and other complications after taking sacubitril/valsartan, suggesting that the mentioned therapy is safe to a certain degree.

## Conclusions

In conclusion, we reported the successful use of sacubitril/valsartan in a patient with CRS4 and stage 5 CKD, in which NYHA class IV reduced to NYHA class II, the levels of NT-proBNP, LAD, LVID, and CTR decreased, LVEF increased, blood pressure attenuated, and renal function stable after more than 3 years of follow-up.

## Data Availability Statement

The original contributions presented in the study are included in the article/supplementary material, further inquiries can be directed to the corresponding author.

## Ethics Statement

The studies involving human participants were reviewed and approved by the Local Ethics Committee of Jinling Hospital. The patients/participants provided their written informed consent to participate in this study. Written informed consent was obtained from the individual(s) for the publication of any potentially identifiable images or data included in this article.

## Author Contributions

YY, SC, and TZ participated in research design. SC, TZ, and LY participated in the writing of the article. YC, ZZ, and JW performed revision of the manuscript. All authors read and approved the final version.

## Funding

This study was financially supported by the Jiangsu Clinical Medical Center (Grant No. YXZXA2016003).

## Conflict of Interest

The authors declare that the research was conducted in the absence of any commercial or financial relationships that could be construed as a potential conflict of interest.

## Publisher's Note

All claims expressed in this article are solely those of the authors and do not necessarily represent those of their affiliated organizations, or those of the publisher, the editors and the reviewers. Any product that may be evaluated in this article, or claim that may be made by its manufacturer, is not guaranteed or endorsed by the publisher.

## References

[1] Haynes R Zhu D Judge PK Herrington WG Kalra PA Baigent C. Chronic kidney disease, heart failure and neprilysin inhibition. Nephrol Dial Transplant. (2020) 35:558–64. 10.1093/ndt/gfz058 31028383 PMC7139204

[2] Chuppa S Liang M Liu P Liu Y Casati MC Cowley AW. MicroRNA-21 regulates peroxisome proliferator-activated receptor alpha, a molecular mechanism of cardiac pathology in Cardiorenal Syndrome Type 4. Kidney Int. (2018) 93:375–89. 10.1016/j.kint.2017.05.014 28760335 PMC5787401

[3] Pinheiro Da Silva AL Vaz Da Silva MJ. Type 4 cardiorenal syndrome. Rev Port Cardiol. (2016) 35:601–16. 10.1016/j.repc.2016.06.007 27712930

[4] Sabbah HN Zhang K Gupta RC Xu J Singh-Gupta V. Effects of angiotensin-neprilysin inhibition in canines with experimentally induced cardiorenal syndrome. J Card Fail. (2020) 26:987–97. 10.1016/j.cardfail.2020.08.009 32841710 PMC7704862

[5] Vasquez N Carter S Grodin JL. Angiotensin receptor-neprilysin inhibitors and the natriuretic peptide axis. Curr Heart Fail Rep. (2020) 17:67–76. 10.1007/s11897-020-00458-y 32394149

[6] Kang G Banerjee D. Neprilysin inhibitors in cardiovascular disease. Curr Cardiol Rep. (2017) 19:16. 10.1007/s11886-017-0827-0 28185171

[7] Raphael DM Liu Z Jin Z Cui X Han D He W. Effects of Sacubitril/Valsartan on clinical symptoms, echocardiographic parameters, and outcomes in HFrEF and HFmrEF patients with coronary heart disease and chronic kidney disease. Curr Med Res Opin. (2021) 37:1071–8. 10.1080/03007995.2021.1908243 33764230

[8] Damman K Gori M Claggett B Jhund PS Senni M Lefkowitz MP. Renal effects and associated outcomes during angiotensin-neprilysin inhibition in heart failure. JACC Heart Fail. (2018) 6:489–98. 10.1016/j.jchf.2018.02.004 29655829

[9] Matsushita K Selvin E Bash LD Astor BC Coresh J. Risk implications of the new CKD Epidemiology Collaboration (CKD-EPI) equation compared with the MDRD Study equation for estimated GFR: the Atherosclerosis Risk in Communities (ARIC) Study. Am J Kidney Dis. (2010) 55:648–59. 10.1053/j.ajkd.2009.12.016 20189275 PMC2858455

[10] Ricci Z Romagnoli S Ronco C. Cardiorenal syndrome. Crit Care Clin. (2021) 37:335–47. 10.1016/j.ccc.2020.11.003 33752859

[11] Okabe T Yakushiji T Kido T Kimura T Asukai Y Shimazu S. Poor prognosis of heart failure patients with in-hospital worsening renal function and elevated BNP at discharge. ESC Heart Fail. (2020) 7:2912–21. 10.1002/ehf2.12901 32643875 PMC7524072

[12] Gori M. D'elia E, Senni M. Sacubitril/valsartan therapeutic strategy in HFpEF: clinical insights and perspectives. Int J Cardiol. (2019) 281:158–65. 10.1016/j.ijcard.2018.06.060 30420146

[13] Chen Y Guo LY Zhao LF Ma YH Zhu XL Xu Y. Can sacubitril/valsartan become the promising drug to delay the progression of chronic kidney disease? J Geriatr Cardiol. (2020) 17:782–6. 10.11909/j.issn.1671-5411.2020.12.002 33424946 PMC7762693

[14] Mcmurray JJ Packer M Desai AS Gong J Lefkowitz MP Rizkala AR. Angiotensin-neprilysin inhibition versus enalapril in heart failure. N Engl J Med. (2014) 371:993–1004. 10.1056/NEJMoa1409077 25176015

[15] Januzzi JL Jr. Prescott MF Butler J Felker GM Maisel AS Mccague K. Association of change in N-Terminal Pro-B-Type natriuretic peptide following initiation of sacubitril-valsartan treatment with cardiac structure and function in patients with heart failure with reduced ejection fraction. JAMA. (2019) 322:1085–95. 10.1001/jama.2019.12821 31475295 PMC6724151

[16] De Diego C Gonzalez-Torres L Nunez JM Centurion Inda R Martin-Langerwerf DA Sangio AD. Effects of angiotensin-neprilysin inhibition compared to angiotensin inhibition on ventricular arrhythmias in reduced ejection fraction patients under continuous remote monitoring of implantable defibrillator devices. Heart Rhythm. (2018) 15:395–402. 10.1016/j.hrthm.2017.11.012 29146274

[17] Solomon SD Mcmurray JJV Anand IS Ge J Lam CSP Maggioni AP. Angiotensin-neprilysin inhibition in heart failure with preserved ejection fraction. N Engl J Med. (2019) 381:1609–20. 10.1056/NEJMoa1908655 31475794

[18] Nie D Xiong B Qian J Rong S Yao Y Huang J. The effect of sacubitril-valsartan in heart failure patients with mid-range and preserved ejection fraction: a meta-analysis. Heart Lung Circ. (2021) 30:683–91. 10.1016/j.hlc.2020.10.012 33199181

[19] Volpe M Bauersachs J Bayes-Genis A Butler J Cohen-Solal A Gallo G. Sacubitril/valsartan for the management of heart failure: a perspective viewpoint on current evidence. Int J Cardiol. (2021) 327:138–45. 10.1016/j.ijcard.2020.11.071 33301829

[20] Beltrán P Palau P Dominguez E Faraudo M Nunez E Guri O. Sacubitril/valsartan and short-term changes in the 6-minute walk test: a pilot study. Int J Cardiol. (2018) 252:136–9. 10.1016/j.ijcard.2017.10.074 29249422

[21] Selvaraj S Claggett BL Bohm M Anker SD Vaduganathan M Zannad F. Systolic blood pressure in heart failure with preserved ejection fraction treated with sacubitril/valsartan. J Am Coll Cardiol. (2020) 75:1644–56. 10.1016/j.jacc.2020.02.009 32192799 PMC8109279

[22] Kang H Zhang J Zhang X Qin G Wang K Deng Z. Effects of sacubitril/valsartan in patients with heart failure and chronic kidney disease: a meta-analysis. Eur J Pharmacol. (2020) 884:173444. 10.1016/j.ejphar.2020.173444 32739172

[23] Wang TD Tan RS Lee HY Ihm SH Rhee MY Tomlinson B. Effects of Sacubitril/Valsartan (LCZ696) on natriuresis, diuresis, blood pressures, and NT-proBNP in salt-sensitive hypertension. Hypertension. (2017) 69:32–41. 10.1161/HYPERTENSIONAHA.116.08484 27849566

[24] Martens P Nuyens D Rivero-Ayerza M Van Herendael H Vercammen J Ceyssens W. Sacubitril/valsartan reduces ventricular arrhythmias in parallel with left ventricular reverse remodeling in heart failure with reduced ejection fraction. Clin Res Cardiol. (2019) 108:1074–82. 10.1007/s00392-019-01440-y 30788621

[25] Abumayyaleh M El-Battrawy I Behnes M Borggrefe M Akin I. Current evidence of sacubitril/valsartan in the treatment of heart failure with reduced ejection fraction. Future Cardiol. (2020) 16:227–36. 10.2217/fca-2020-0002 32186406

[26] El-Battrawy I Akin I. Impact of Sacubitril/Valsartan on cardiac arrest event rate. Eur J Heart Fail. (2022). 10.1002/ejhf.2444 35118775

[27] El-Battrawy I Pilsinger C Liebe V Lang S Kuschyk J Zhou X. Impact of sacubitril/valsartan on the long-term incidence of ventricular arrhythmias in chronic heart failure patients. J Clin Med. (2019) 8:1582. 10.3390/jcm8101582 31581623 PMC6832713

[28] El-Battrawy I Borggrefe M Akin I. The risk for sudden cardiac death and effect of treatment with sacubitril/valsartan in heart failure. JACC Heart Fail. (2019) 7:999. 10.1016/j.jchf.2019.05.010 31672315

[29] Ambrosy AP Braunwald E Morrow DA Devore AD Mccague K Meng X. Angiotensin receptor-neprilysin inhibition based on history of heart failure and use of renin-angiotensin system antagonists. J Am Coll Cardiol. (2020) 76:1034–48. 10.1016/j.jacc.2020.06.073 32854838

[30] Uijl E. T Hart DC Roksnoer LCW Groningen MCC Van Veghel R Garrelds IM. Angiotensin-neprilysin inhibition confers renoprotection in rats with diabetes and hypertension by limiting podocyte injury. J Hypertens. (2020) 38:755–64. 10.1097/HJH.0000000000002326 31790054

[31] Habibi J Aroor AR Das NA Manrique-Acevedo CM Johnson MS Hayden MR. The combination of a neprilysin inhibitor (sacubitril) and angiotensin-II receptor blocker (valsartan) attenuates glomerular and tubular injury in the Zucker Obese rat. Cardiovasc Diabetol. (2019) 18:40. 10.1186/s12933-019-0847-8 30909895 PMC6432760

[32] Mohany M Alanazi AZ Alqahtani F Belali OM Ahmed MM. and Al-Rejaie SS. LCZ696 mitigates diabetic-induced nephropathy through inhibiting oxidative stress, NF-kappaB mediated inflammation and glomerulosclerosis in rats. PeerJ. (2020) 8:e9196. 10.7717/peerj.9196 32596035 PMC7307563

[33] Pitt B Rossignol P. Impact of hyperkalemia and worsening renal function on the use of renin angiotensin aldosterone system inhibitors in chronic heart failure with reduced ejection fraction. Clin Pharmacol Ther. (2017) 102:389–91. 10.1002/cpt.746 28707341

[34] Masarone D Melillo E Errigo V Valente F Pacileo G. Clinical relevance of transient worsening renal function after initiation of sacubitril/valsartan. Curr Med Res Opin. (2021) 37:9–12. 10.1080/03007995.2020.1853509 33210952

